# Sequential and counter-selectable cassettes for fission yeast

**DOI:** 10.1186/s12896-016-0307-4

**Published:** 2016-11-08

**Authors:** Hanna Amelina, Vera Moiseeva, Laura Catharine Collopy, Siân Rosanna Pearson, Christine Anne Armstrong, Kazunori Tomita

**Affiliations:** Chromosome Maintenance Group, UCL Cancer Institute, University College London, Paul O’Gorman Building, Huntley Street, London, WC1E 6DD UK

**Keywords:** Schizosaccharomyces pombe, DNA replication, Point mutation, Gene disruption and insertion, Thymidine kinase, FUdR, HA, Flag, PK tagging, Zeocin

## Abstract

**Background:**

Fission yeast is one of the most commonly used model organisms for studying genetics. For selection of desirable genotypes, antibiotic resistance cassettes are widely integrated into the genome near genes of interest. In yeasts, this is achieved by PCR amplification of the cassette flanked by short homology sequences, which can be incorporated by homology directed repair. However, the currently available cassettes all share the same *tef* promoter and terminator sequences. It can therefore be challenging to perform multiple genetic modifications by PCR-based targeting, as existing resistance cassettes in strains can be favored for recombination due to shared homology between the cassettes.

**Results:**

Here we have generated new selection cassettes that do not recombine with those traditionally used. We achieved this by swapping the *tef* promoter and terminator sequences in the established antibiotic resistance MX6 cassette series for alternative promoters and/or terminators. The newly created selection cassettes did not recombine with the *tef*-containing MX6 cassettes already present in the genome, allowing for sequential gene targeting using the PCR-based method. In addition, we have generated a series of plasmids to facilitate the C-terminal tagging of genes with desired epitopes. We also utilized the anti-selection gene *HSV-TK*, which results in cell death in strains grown on the drug 5-Fluoro-2’-deoxyuridine (FdU, Floxuridin or FUDR). By fusing an antibiotic resistance gene to *HSV-TK,* we were able to select on the relevant antibiotic as well as counter-select on FdU media to confirm the desired genomic modification had been made. We noted that the efficiency of the counter selection by FdU was enhanced by treatment with hydroxyurea. However, a number of DNA replication checkpoint and homologous recombination mutants, including *rad3*∆, *cds1*∆, *rad54*∆ and *rad55*∆, exhibited sensitivity to FdU even though those strains did not carry the *HSV-TK* gene. To remove counter-selectable markers, we introduced the Cre-loxP irreversible recombination method. Finally, utilizing the negative selectable markers, we showed efficient induction of point mutations in an endogenous gene by a two-step transformation method.

**Conclusions:**

The plasmid constructs and techniques described here are invaluable tools for sequential gene targeting and will simplify construction of fission yeast strains required for study.

## Background

Fission yeast *Schizosaccharomyces pombe* is a popular model organism for fundamental biological research [1]. A haploid life cycle along with efficient homologous recombination system and simple reproductive processes make it an excellent system to study genetics. In yeast, genes can be deleted and replaced with selection markers readily, simply by inducing homology directed repair with a selection cassette amplified with primers that contain short, flanking sequences homologous to a target gene [2, 3]. A strain of induced genotype can be isolated simply by plating transformants onto corresponding selection plates. Generated strains can be further crossed to isolate multiple genetic alternations that can be selected for using combination of appropriate markers.

In fission yeast, commonly used drug selection cassettes are *kanMX6*, *natMX6*, *hygMX6* (*hphMX6*) and *bleMX6* [2, 4, 5]. These cassettes are composed of a promoter and terminator from the fungus *Ashbya gossypii* and genes conferring selectable resistance to G418 (geneticin or neomycin), nourseothricin (ClonNat or Nat), hygromycin B and bleomycin respectively. Auxotrophic gene cassettes, such as *ura4* ^*+*^ from *S. pombe* and *LEU2* from *Saccharomyces cerevisiae* have also been extensively used in fission yeast [6, 7]. However, when using auxotrophic cassettes, strains must have mutations in the corresponding endogenous gene, which could affect cell metabolism, stress response and cell growth. Thus, use of antibiotic resistance markers has become standard for yeast organisms, as the working strain does not require any particular auxotrophic genetic background. However, as these antibiotic cassettes all share the same promoter and terminator sequences, it is difficult to perform sequential gene targeting due to the shared homology [5]. This issue is usually solved by genetic cross between two mutant strains. However, this strategy is not feasible for mutants dysfunctional in mating, meiosis or germination. In some cases the mating is successful, but it is difficult to obtain desired genotype in the offspring if genetic distance is too close or if desired genotypes are not associated with selectable marker or not readily isolated by the marker. Importantly, genetic crossing also mixes epigenetic status of genome. For example, telomere length will change if strains harboring distinct mean length of telomeres are crossed. These risks are avoidable when sequential gene targeting is performed. Therefore, *ura4* ^+^, *arg3* ^+^ and *his3* ^+^ and *LEU2* cassettes are still used, as these auxotrophic markers contain their own distinct promoters and terminators [6–9].

The recently developed genome editing technology, CRISPR (Clustered regularly interspaced short palindromic repeats) and Cas9 (CRISPR-associated protein-9 endonuclease), has become available to use in fission yeast [10]. In this system, the Cas9 nuclease is targeted to specific regions of genomic DNA by its CRISPR RNA component, a so-called guide RNA that binds to the target DNA, resulting in Cas9-mediated DNA double-strand breaks (DSBs) at the target genomic locus. This system does not require selection cassettes, as cells confer constitutive DNA DSBs until the target DNA sequence is lost or altered. Because a selection cassette is not required, this method is ideal for the sequential targeting of multiple genes/loci; in particular for gene disruption and for introducing point mutations or insertions using a designed DNA repair template. However, although the CRISPR/Cas9 strategy is widely used for genetic engineering in animals and humans, the use of this method to mutate genes in fission yeast currently holds a number of limitations. It is often necessary to design a number of guide RNAs, which need to be cloned into a fission yeast CRISPR/Cas9 plasmid [10]. Target sequences must be unique, 20 bases long, and are limited to loci upstream of a protospacer adjacent motif (−NGG for Cas9). This means that the designed DNA repair template will recombine only at the DSB site, and the substitution of the designed point mutation needs to be close to the DSB site [11]. It is also noted that adenine and thymine rich sequences cannot be targeted. Hence, whereas gene disruption, single point mutations and insertions are relatively straightforward for the CRISPR/Cas9 method, replacement of a large genomic DNA region with the designed DNA template, such as wide range multiple mutagenesis of the whole gene sequence [12], is not feasible. Thus, traditional homologous recombination-based genetic engineering remains the most robust method to perform genetic manipulations in fission yeast.

Here, we report optimization of the classical PCR-based targeting method by modification of well-known *MX6* markers, which enables sequential knock in/out genes of interest. We also generated the C-terminal tagging vectors for PCR-based gene targeting. In addition, we generated counter selectable drug resistance markers by fusing the herpes simplex virus thymidine kinase (HSV-TK) with antibiotic resistance genes. This can replace the traditionally used counter-selectable *ura4* ^+^ cassette. Furthermore, introduction of irreversible mutant *loxP* (locus of X(cross)-over P1) sites allows recycling of selection cassettes. Finally, using the generated constructs, we report a reliable two-step mutagenesis method, suitable for mutagenesis of essential genes. Altogether, we have demonstrated innovative techniques that address current difficulties in the construction of required fission yeast strains.

## Results and discussion

### Cassettes for PCR-based sequential targeting

**Fig. 1:**
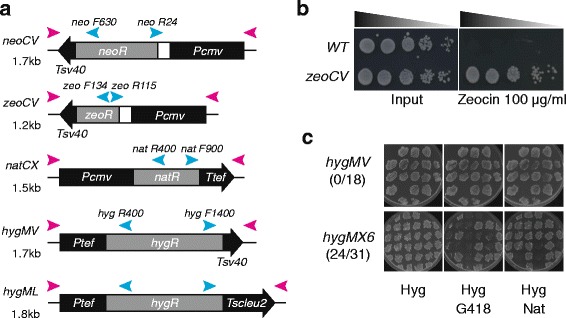
Selection markers for sequential gene targeting. **a** Schematic representation of sequential targeting cassettes pFA6a-neoCV, pFA6a-zeoCV, pFA6a-natCX, pFA6a-hygMV and pFA6a-hygML. The region used as a PCR template is shown. The 100 base *Top (Tag)* primer anneals to the left end (*pink arrowhead on the left*) and *Bot* primer anneals to the right end to amplify indicated cassettes (*pink arrowheads on the right*). The 100 base primers used in this study are listed in Table 1. Cyan arrows represent diagnostic primers used for screening of correct targeting (Table 2). Sequences of primers are shown in Table 2. Backbone vector region (pFA6a) encodes ampicillin resistant cassette and *ColE1* bacteria replication origin, and the cassettes were inserted between *Pac*I and *Pme*I sites. *Black box* and *black arrow* in the cassette represent indicated promoter and terminator, respectively. *White box* in *neoCV* and *zeoCV* indicates the *em7* promoter for *E. coli*. The size of the cassette is shown on the left. Transcription direction is toward left for *neoCV* and *zeoCV* cassettes and toward right for other cassettes. **b** Wild-type and *rdh54*∆ cells in which the *zeoCV* cassette replaced *rdh54* were cultured in YES rich media, normalised and serially diluted. Five microliter of diluted fractions were spotted on YES (input) or YES containing 100 μg/ml of Zeocin^TM^ and incubated at 32 °C for 3 days. Only cells containing the *zeoCV* cassette grew on YES with Zeocin. Deletion of the *rdh54* gene required for meiosis does not impair cell growth and mitotic DNA damage repair. **c** Cells carrying *kanMX6* or *natMX6* cassettes were transformed with *hygMV* (Top) or *hygMX6* (*Bottom*) and selected for on YES plates containing 100 μg/ml hygromycin (Hyg). Eighteen and 31 colonies that were randomly picked for *hygMV* and *hygMX6 transformations respectively,* were re-streaked on YES plates containing 100 μg/ml Hyg (*Left panel*). Cells were then replica-plated to YES plates containing 100 μg/ml Hyg plus either 100 μg/ml G418 (*Middle panel*) or 100 μg/ml ClonNat (Nat) (*Right panel*). In case of *hygMV* transformation, all transformed cells retained resistance to G418 and ClonNat, whereas in case of *hygMX6, only seven out of 31 did*

Although PCR-based gene targeting by drug selection markers is the conventional approach, it is not efficient if a transforming strain already contains other drug selection markers. All *MX6* cassettes share 300 bp of the *tef* promoter and 200 bp of the *tef* terminator, which are significantly larger than the 80–100 bp sequences introduced in primers to target genes of interest. Therefore, there is a high chance of recombination between the *MX6* cassettes [5]. In order to overcome this problem, *CMVneo* and *CMVzeo* cassettes (Life Technology) were cloned to replace the *MX6* cassette of the plasmid pFA6a-kanMX6 [2]. Resulting plasmids, pFA6a-neoCV and pFA6a-zeoCV, contain neomycin/kanamycin and Zeocin^TM^ resistant genes, respectively, flanked by the cytomegalovirus promoter (*Pcmv*) and the simian virus 40 terminator (*Tsv40*) (Fig. 1a). These cassettes share only 20 bases of the primer annealing sites with the MX6 cassettes, which reduces the risk of unwanted recombination between selection markers. In addition, the *tef* promoter in the *natMX6* cassette, which provides resistance to nourseothricin (Nat), was replaced with the *CMV* promoter (*Pcmv*), resulting in the plasmid named pFA6a-natCX. The *tef* terminator of the *hygMX6* cassette, which provides resistance to Hygromycin B (Hyg), was replaced with either the *Tsv40* terminator or the *S. cerevisiae LEU2* terminator (*Tscleu2),* resulting in the plasmids named pFA6a-hygMV and pFA6a-hygML, respectively (Fig. 1a). The *zeoCV* cassette acts as an equivalent to *bleMX6* [4], and provides resistance to Zeocin^TM^, bleomycin and phleomycin (Fig. 1b). We confirmed that replacement of the promoter or terminator did not impair the function of the drug resistant cassettes. Thus, the newly generated selection cassettes can be used as an alternative to existing MX6 cassettes.

Using the generated cassettes, we tested if they could be used for sequential gene deletion. Two genes, *crb2* ^+^ and *rdh54* ^+^, were sequentially targeted in strains bearing both a *kanMX6* and *natMX6* cassette. Crb2 is the fission yeast ortholog of human 53BP1 [13], and Rdh54 is the equivalent of Rad54, which is required for meiotic recombination [14]. To delete *crb2* ^*+*^ *,* the *hygMV* cassette was amplified by PCR using the *crb2 100 bp* primer set (Table 1, crb2 top and bot) and pFA6a-hygMV plasmid as a template. A strain bearing both *kanMX6* and *natMX6* [*h* ^*90*^ *ura4-D18 taz1-YFP:kanMX6 sid4-mCherry:natMX6 hht1-Cerulean:ura4* ^*+*^] was transformed with the *crb2::hygMV* PCR fragment, and the transformant colonies were selected for resistance to hygromycin B (Hyg). The presence of *kanMX6* and *natMX6* cassettes was determined by resistance to G418 and ClonNat (Nat), respectively. The *hygMV* cassette was therefore successfully integrated into the genome without recombining with the MX6 cassettes present, conferring resistance to multiple antibiotics (Fig. 1c: hygMV panel). Deletion of *crb2* by *hygMV* was confirmed by diagnostic PCR using a forward primer within the *hyg* ^*R*^ gene and a reverse primer downstream *of crb2* (for sequences see Table 2, *hyg F1400* and *crb2 R280D)*. In contrast, transformation with the *crb2::hygMX6* PCR fragment resulted in replacement of *kanMX* or *natMX6* markers, assessed by gain of sensitivity to G418 and Nat, respectively (Fig. 1c: hygMX6 panel). Next, the *rdh54* ^+^ gene was deleted in a similar fashion, using a *rdh54::zeoCV* fragment generated by PCR using the *rdh54 100 bp* primer set and pFA6a-zeoCV plasmid as a template. Like *hygMV*, *zeoCV* did not recombine with any of existent marker cassettes (data not shown). The *rdh54* deletion in the resulting strain [*h* ^*90*^ *ura4-D18 crb2::hygMV rdh54::zeoCV taz1-YFP:kanMX6 sid4-mCherry:natMX6 hht1-Cerulean:ura4* ^*+*^] was confirmed by diagnostic PCR using primers *rdh54 R265D* and *zeo R115* (Table 2). We also confirmed that *neoCV*, *hygML* and *natCX* did not recombine with the MX6 cassettes (data not shown). Therefore, we conclude that replacement of the *tef* promoter and/or terminator sequences prevents recombination of newly introduced drug selection cassette with the MX6 cassettes already existing in a strain.

**Table 1:** One hundred base primers for PCR-based targeting fragments used in this study

Name	Sequence
*crb2 Top*	CCCTGGTTAAATTTGTAGTTCTGACAATTGTGAGGTATTTTAGATGTTTTCAATATTTTGTTTGAAAGTTTAACAATATTCGGATCCCCGGGTTAATTAA
*crb2 Bot*	CTAAAATTAATAAAAAGCTAAATTAATGAGAGTGAAACTCAGGGGGAGTTAGTAAAAATAACTATATCAAAAAACCAAAAGAATTCGAGCTCGTTTAAAC
*rdh54 Top*	ACTTGAACACCACACGTTCGGTCTCATAGTATTGTTGAGTAAATAAACACAGTTACCAAAGAGAATTGAAACCTTACTTTCGGATCCCCGGGTTAATTAA
*rdh54 Bot*	TGCTCATAGAGATTGCCCAGTATCGAAGGCTTGCTCAAAAGCGTTGTTTCTAATGGGCAAGGAAAAACCGCTTACGCCTCGAATTCGAGCTCGTTTAAAC
*tpz1 Tag*	CTCTGAGGCCTGTGAAATGTGTCGGCTTGGGCTACCTCATGGATCATTCTTTGAGCTATTGCGAGATTGGAAAAAAATAGAGGAGTTTCGAAACAAAAGCCGGATCCCCGGGTTAATTAA
*tpz1 Bot*	TTGGTCCGTTGTAAGCCATTTCACTGTATGTCTGTAACAGTTAACTTCCGTACTTAGTAAAATGTTAGTAAAAAAGGAAGATATGTGATACAGCAATTGAGAATTCGAGCTCGTTTAAAC
*tpz1 Top*	ATCAACAGACTTCAGTCAGCACTGTTACTTATTAAAAAAAGTTGATTTTTATATAAAAGTTAGCTGCGTTAAACAGTGCACGGATCCCCGGGTTAATTAA

Under line encodes annealing site for the template. Top primer contains CGGATCCCCGGGTTAATTAA at the 3’ end and 80–100 base upstream sequence of the target site at the 5’ end. Bot primer contains GAATTCGAGCTCGTTTAAAC at the 3’ end and 80–100 base downstream complement sequence of the target site at the 5’ end

**Table 2:** Primers for diagnostic PCR

Name	Sequence
*neo R24*	GTGCAATCCATCTTGTTCAATC
*neo F630*	TTTCTGGATTCATCGACTGTGG
*zeo R115*	CGAAGTCGTCCTCCACGAAG
*zeo F134*	ACGTGACCCTGTTCATCAGC
*nat F900*	GGGGTTCACCCTCTGCGGCC
*nat R400*	GGGACACTGGTGCGGTACCG
*hyg F1400*	CCGTCTGGACCGATGGCTGT
*hyg R400*	CTTCTCGACAGACGTCGCGG
*kan F800*	GGATTCAGTCGTCACTCATGGTG
*kan R276*	ATGCATCATCAGGAGTACGG
*TK F1040*	TACCGACGATCTGCGACCTG
*TK R90*	ACTTCCGTGGCTTCTTGCTG
*adh1 R540U*	AGGAATACGGATACGATGGAG
*Ptef R81*	ACATGGGGATGTATGGGCTA
*Ttef F1*	CAGTACTGACAATAAAAAGATTCTTG
*crb2 R280D*	CAACCATTCGAAACCTGCTAC
*rdh54 R265D*	GAAAGCCAGAAACAGACAAGC
*tpz1 F842U*	GGTAGTAGGCTAAATGTGAGTTG
*tpz1 R400D*	CCCTTAGAAGATAAGCTCAACC

### HSV-TK chimera selection markers

Until recently, the *ura4* ^+^ auxotrophic marker was the only marker in the fission yeast system to be both selectable and counter-selectable. Cells carrying *ura4* ^+^ gene can be eliminated by growing on media supplemented with 5-Fluoroorotic acid (5-FOA), as Ura4 converts 5-FOA to toxic fluorodeoxyuridine [7]. Alternatively, the thymidine kinase from herpes simplex virus (HSV-TK, or TK) is available for use as a negative selection marker [15]. TK phosphorylates 5-Fluoro-2’-deoxyuridine (FdU, Floxuridin or FUDR) to give 5-Fluoro-2’-deoxyuridine-5-monophosphate (FdUMP). FdUMP blocks activity of thymidylate synthase, resulting in inhibition of 2’-deoxythymidine-5-triphosphate (dTTP) synthesis, leading to loss of the dTTP pool and cell death. Hence, strains expressing a *TK* cassette cannot grow in the presence of FdU [15, 16]. Unlike the *ura4* ^+^ cassette, the *TK* cassette can be used with any genotype background. Although fission yeast carrying the *TK* cassette are sensitive to FdU, a positive selectable marker is also needed in order to isolate cells carrying *TK* [15]. This issue was solved by a fusion of the *TK* gene with a positive selectable marker in mammalian systems [17, 18].

**Fig. 2:**
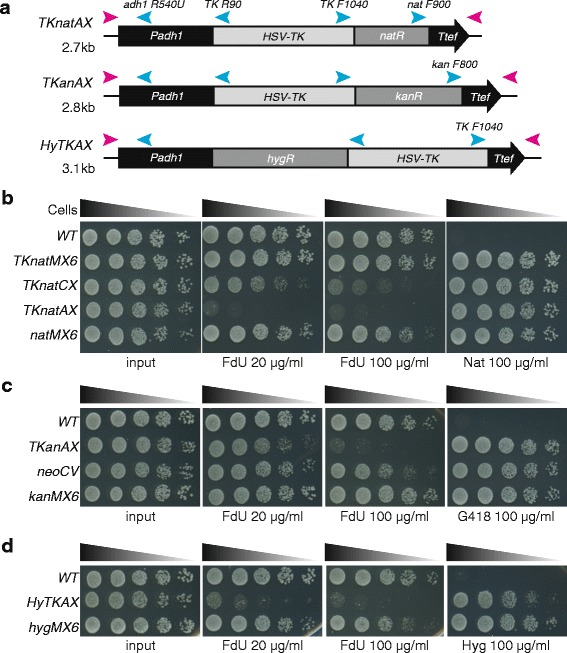
TK-fusion counter-selectable cassettes. **a** Schematic representation of counter-selectable cassettes *HyTKAX*, *TKanAX* and *TKnatAX*. Region used as a PCR template is shown. The backbone vector is pFA6a and the cassettes were inserted between *Pac*I *Pme*I sites. *Top* primers anneal to the left end (*pink arrowhead on the left*) and *Bot* primer anneals to the right end to amplify indicated cassettes (*pink arrowheads on the right*). *Cyan arrows* represent diagnostic primers used for screening of correct targeting, and sequence of primers is shown in Table 2. The *TK* fusion genes are transcribed by the *S. pombe adh1* promoter and terminated at the *tef* terminator. The size of the cassette is shown on the left. Transcription direction is toward right. **b**, **c** and **d** FdU sensitivity of cells carrying the *HSV-TK* fusion cassettes. Indicated cells were cultured in YES rich media, normalised and sequentially diluted five times. Five microliter of diluted fractions were spotted on YES containing the indicated drugs and incubated at 32 °C for 3 days. **b** Cells carrying the *TKnatAX* cassette are hyper sensitive to 20 μl/ml FdU. **c** Cells carrying the *TKanAX* cassette are sensitive to 100 μl/ml FdU. **d** Cells carrying the *HyTKAX* cassette are sensitive to 20–100 μl/ml FdU

We decided to create fused selection markers for fission yeast, which express fusion antibiotic resistance genes with the *TK* gene that serve for both positive and negative selection by chemical treatments. We first inserted the *TK* gene upstream of the gene coding sequences in *kanMX6* and *natMX6*, giving *TKanMX6* and *TKnatMX6*. To assess function of the fusion genes, the TK-fusion cassettes were inserted at the *leu1* ^+^ locus. As expected, cells carrying *TKanMX6* and *TKnatMX6* were resistant to G418 and Nat, respectively. However, these cells grew in the presence of 100 μg/ml FdU, suggesting that the fused *TK* gene is not functional (see the *TKnatMX6* in Fig. 2b). We speculated that the level of TK, expressed by the *tef* promoter, was not sufficient to convert FdU for a toxic amount of FdUMP, as previously published the *TK* cassette for fission yeast uses the stronger *adh1* promoter [15]. We therefore replaced the *tef* promoter with a stronger *CMV* promoter (giving *TKnatCX*, Fig. 2a), which indeed enhanced cells sensitivity to FdU (Fig. 2b). Furthermore, an even greater sensitivity was obtained with replacement of the *S. pombe adh1* promoter, giving *TKnatAX* (Fig. 2a and b). The success of this finding was reiterated with the *TK-kan* ^*R*^ fusion gene, giving *TKanAX* (Fig. 2a). Cells carrying *TKanAX* were fully resistant to G418 and displayed an intermediate sensitivity to FdU (Fig. 2c). Finally, *HSV-TK* was inserted after the *hyg* ^*R*^ gene coding sequence, similar to the *HyTK or HygR-TK* fusion genes used in mammalian systems [17], giving *HyTKAX* (Fig. 2a). Cells carrying *HyTKAX* were resistant to hygromycin B and sensitive to FdU (Fig. 2d). In conclusion, fusions of the yeast antibiotic markers with the *TK* gene are functional, allowing both positive and negative selections of cells carrying the fusion cassettes.

**Fig. 3:**
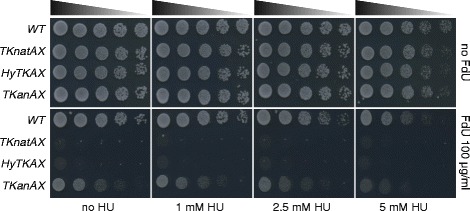
Concomitant treatment with HU enhances sensitivity to FdU. Indicated cells were cultured in YES, normalised and serially diluted five times with YES. Five microliter of diluted fractions were spotted on rich media containing the indicated drugs and incubated at 32 °C for 3 days. Cells carrying *TKanAX* grew poorly in YES containing 5 mM HU and 100 μg/ml FdU

In yeast, low sensitivity to FdU is partially caused by poor uptake of thymidine. This problem can be overcome by ectopic expression of human equilibrative nucleoside transporter (ENT), which is highly efficient at the uptake of thymidine analogs [19]. However, this strategy requires cells to carry the ENT expression cassette. Toxicity of FdU in the presence of TK is caused by impaired deoxythymidine-5-monophosphate (dTMP) synthesis and reduced dTTP levels [16]. Hence, we anticipated that reduction of the deoxynucleotide (dNTP) pool by hydroxyurea (HU) might further reduce the dTTP level, leading to increased sensitivity to FdU. To test this possibility, cells carrying *TKnatAX*, *TKanAX* and *HyTKAX* were spotted on YES media containing increasing concentrations of HU (1, 2.5 and 5 mM) along with 100 μg/ml FdU (Fig. 3). The growth of cells carrying *TKanAX* was significantly suppressed by FdU in the presence of HU, as represented by microcolonies. Taken together, we recommend the following concentrations for FdU anti-selections: 20 μg/ml FdU for *TKnatAX*, 100 μg/ml FdU for *HyTKAX* and 100 μg/ml FdU plus 5 mM HU for *TKanAX*.

### Mutants defective in DNA replication checkpoint and homologous recombination are sensitive to FdU

**Fig. 4:**
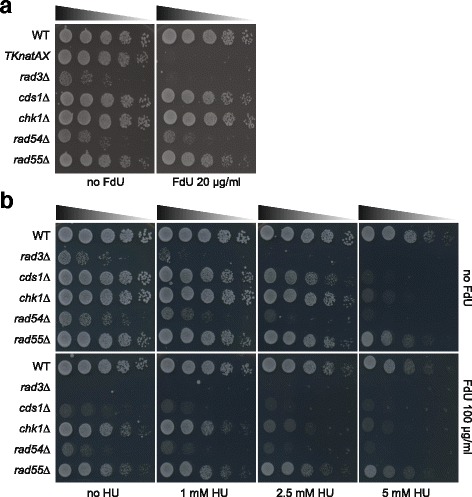
*rad3*∆, *cds1*∆ and *rad54*∆ are sensitive to FdU. Indicated cells were cultured in YES, normalised and 5 times sequentially diluted with YES. Five microliter of diluted fractions were spotted on rich media containing the indicated drugs and incubated at 32 °C for 3 days. **a** *rad3*∆ cells are hyper sensitive to 20 μg/ml FdU. **b** *cds1∆* and *rad54∆* cells are sensitive to 100 μg/ml FdU. *chk1*∆ and *rad55∆* cells are mildly but significantly sensitive to FdU, compared to wild type cells

Although the *TK* cassette is a useful tool, we have come across a number of fission yeast mutants that exhibited sensitivity to FdU while not containing the *TK* cassette. Specifically, our data suggest that mutants defective in DNA replication and DNA damage response pathways are sensitive to FdU. It has previously been reported that *rad3*∆ cells, which lack the DNA damage checkpoint protein Rad3 (a fission yeast ATR homolog [20]), are sensitive to the deoxythymidine variant, 5’-Ethyl-2’-deoxyuridine in the presence of TK [21]. Surprisingly, we identified that *rad3*∆ cells that did not express TK were also sensitive to 20 μg/ml FdU (Fig. 4a). In response to DNA damage and DNA replication problems, Rad3 activates Chk1 and Cds1, respectively [20]. Interestingly, *cds1*∆ cells were sensitive to 100 μg/ml FdU, whereas *chk1*∆ cells were only mildly sensitive to FdU (Fig. 4b). Moreover, homologous recombination repair mutant *rad54*∆ cells also exhibited sensitivity to 100 μg/ml FdU (Fig. 4b). Whilst Rad54 is essential for homologous recombination, the Rad55-Rad57 complex acts in parallel to Swi5-Sfr1 and upstream of Rad54 [22]. As such, *rad55*∆ cells exhibited only mild sensitivity. Although it remains unclear how FdU can be converted to FdUMP under DNA replication stress, our data suggest that mutants defective in DNA replication and DNA damage response pathways are sensitive to FdU. Therefore, we recommend to test working strains for the sensitivity to FdU prior to performing counter-selection screening.

### The Cre/loxP system can be utilized for the recycling of counter-selectable markers

**Fig. 5:**
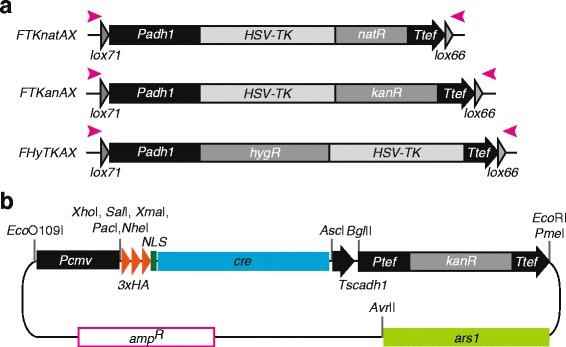
Cre expression vector and loxP cassette. **a** Schematic representation of *loxP*-flanked (floxed) TK-fusion cassettes *FHyTKAX*, *FTKanAX* and *FTKnatAX*. Region used as a PCR template is shown. Forward direction of *lox71* and *lox66* sequences are inserted into the *TK* cassettes shown in Fig. 2a. **b** Schematic representation of pNXRVa-HACre. Fusion protein of three tandem HA epitope tag, NLS (nuclear localization signal) and Cre recombinase is expressed by the constitutively active *CMV* promoter. *kanMX6* is inserted between *Bgl*II and *Pme*I sites. The genomic DNA fragment containing the early replication origin *ARS1* is inserted downstream of the *kanMX6* cassette. A number of unique restriction enzyme sites are indicated

Another way to address the limited availability of selection markers is to recycle the cassette using the Cre/loxP approach [23–25]. In this system, Cre recombinase recombines two short *loxP* sequence sites to generate crossover products. Hence, a *loxP*-flanked (floxed) gene of interest or selection marker can be deleted *via* expression of Cre recombinase. To generate conditional pop-out markers for *TKanAX*, *TKnatAX* and *HyTKAX*, mutant variants of *loxP* sequences - *lox71* and *lox66* - were introduced at the start and end of the cassettes. Resulting cassettes were named *FTKanAX*, *FTKnatAX* and *FHyTKAX*, respectively (Fig. 5a, Table 3). Although the *lox71* and *lox66* sequences are substrates for Cre recombinase, the crossover results in the generation of wild type *loxP* and double mutated *loxP* sequences; the latter is no longer recognized by the recombinase [26]. Therefore, the scar of a *lox71*-*lox66* recombined site will not crossover with a newly introduced *loxP*-containing marker in the genome.

**Table 3:** The targeting cassette vectors

Name	Promoter/gene/terminator	Selection/counter
pFA6a-kanMX6	*Ptef/kan* ^*R*^ */Ttef*	G418
pFA6a-neoCV	*Pcmv/neo* ^*R*^ */Tsv40*	G418
pFA6a-zeoCV	*Pcmv/zeo* ^*R*^ */Tsv40*	Zeocin
pFA6a-hygMX6	*Ptef/hph/Ttef*	G418
pFA6a-hygMV	*Ptef/hph/Tsv40*	Hygromycin B
pFA6a-hygML	*Ptef/hph/Tscleu2*	Hygromycin B
pFA6a-natMX6	*Ptef/nat* ^*R*^ */Ttef*	ClonNat
pFA6a-natCX	*Pcmv/nat* ^*R*^ */Ttef*	ClonNat
pFA6a-TKnatCX	*Pcmv/TK-nat* ^*R*^ */Ttef*	ClonNat/FdU
pFA6a-TKnatAX	*Padh/TK-nat* ^*R*^ */Ttef*	ClonNat/FdU
pFA6a-TKanAX	*Padh/TK-kan* ^*R*^ */Ttef*	G418/FdU + HU
pFA6a-HyTKAX	*Padh/hph-TK/Ttef*	Hygromycin B/FdU
pFA6a-FTKnatAX	*lox71-Padh/TK-nat* ^*R*^ */Ttef-lox66*	ClonNat/FdU
pFA6a-FTKanAX	*lox71-Padh/TK-kan* ^*R*^ */Ttef-lox66*	G418/FdU + HU
pFA6a-FHyTKAX	*lox71-Padh/hph-TK/Ttef-lox66*	Hygromycin B/FdU

We also generated a series of the Cre expression vectors, encoding a fusion protein, comprised of the three tandem hemagglutinin (HA) genes, a nuclear localization signal (nls) sequence and Cre recombinase (3xHA-nls-Cre), under the control of a *CMV* promoter (Fig. 5b). To maximize versatility, the Cre expression vector carries a series of the selection markers; *kanMX6*, *TKkanAX*, *hygMX6*, *HyTKAX*, *natMX6*, *TKnatAX* or the *aur* ^*R*^ cassette, which encodes gene resistance to aureobasidin A [27], as well as auxotrophic markers, including *ura4* ^+^, *arg3* ^+^, *leu1* ^+^ and *ade6* ^+^ (Table 4). Altogether, a choice of three ‘floxed’ markers and a series of the Cre expression vectors would maximize the utility for genome engineering.

**Table 4:** The Cre-expression vectors

Name	Marker cassette	Selection
pNXRVa-HACre	*kanMX6*	G418
pNXRVat-HACre	*TKanAX*	G418
pNXRVb-HACre	*hygMX6*	Hygromycin B
pNXRVbt-HACre	*HyTKAX*	Hygromycin B
pNXRVc-HACre	*natMX6*	ClonNat
pNXRVct-HACre	*TKnatAX*	ClonNat
pNXRVd-HACre	*ura4* ^*+*^	-uracil
pNXRVg-HACre	*arg3* ^*+*^	-arginine
pNXRVh-HACre	*leu1* ^*+*^	-leucin
pNXRVj-HACre	*aur1* ^*R*^	Aureobasidin A
pNXRVk-HACre	*ade6* ^*+*^	-adenine

Using the Cre expression vector, the efficiency of the *FTKnatAX* cassette removal was assessed. Cells carrying the *FTKnatAX* cassette were transformed with the Cre expression vector, and were directly plated on FdU plates. Total 162 colonies were formed on the FdU plate after Cre expression, and all were lost the resistance to Nat. Ten clones were randomly selected and their loss of the *FTKnatAX* cassette was confirmed by PCR. Thus, FdU counter selection along with Cre expression efficiently eliminates cells retaining the *FTKnatAX* Cassette.

### Tandem epitopes C-terminal tagging plasmids, pNX3 series

**Fig. 6:**
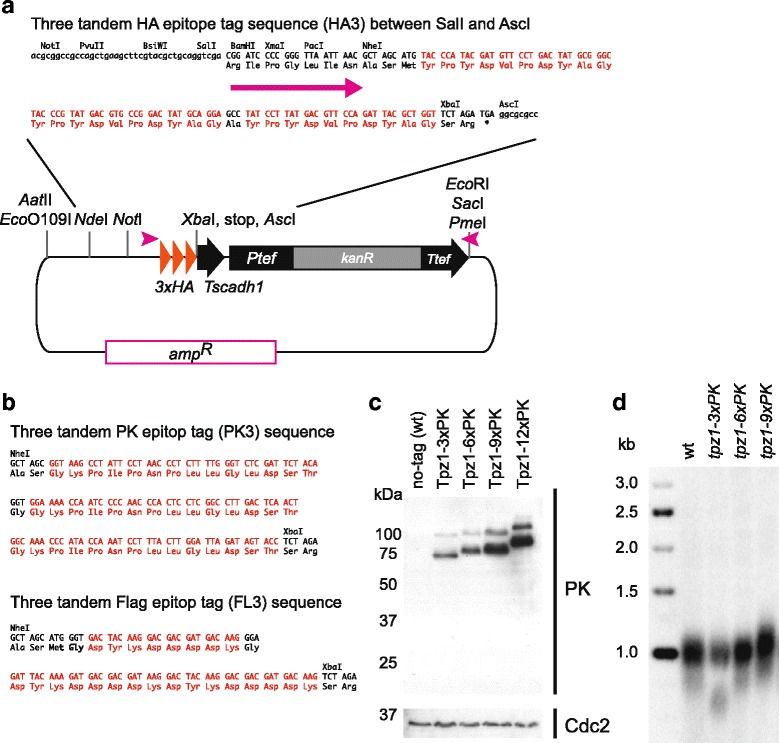
The COOH-terminus tagging plasmid. **a** Schematic representation of pNX3a-HA3 and sequence of three tandem HA and multi cloning sites. The HA encoding gene is inserted between *Pac*I *Asc*I sites. The 100 base *Tag* primer anneals to the left end (*pink arrowhead on the left* in plasmid image and long arrow in HA3 sequence) and *Bot* primer anneals to the right end to amplify indicated cassettes (*pink arrowheads on the right* in plasmid image). A number of unique restriction enzyme sites are indicated. **b** Sequence of three tandem PK (*top*) and FLAG (*bottom*) fragments. pNX3a-PK3 and pNX3a-FL3 plasmids were generated by replacing HA3 sequence between *NheI* and *XbaI* sites in pNX3a-HA3 (**a**) with indicated PK and FLAG sequences, respectively. **c** Detection efficiency of PK-tagged Tpz1. Western blot shows detection of PK epitope fused Tpz1. No obvious non-specific bands were detected. Proteins were extracted from cells and subjected to SDS-PAGE. Anti-V5 antibody (AbD Serotec) was used to detect PK fused Tpz1 protein. Anti-Cdc2 antibody (anti-PSTAIRE) (Santa Cruz) was used as a control for loading. **d** Telomere length homeostasis is slightly impaired with the nine tandem PK tagging of Tpz1. Genomic DNA was harvested from cells cultured over 2 weeks after generation of strains, and digested with *Eco*RI and separated in 1 % agarose gel. Telomere containing fragments were detected with the synthetic telomeric DNA probe

In addition to creating a series of plasmids useful for the sequential modification of genes, we have also created similar plasmids for C-terminal tagging of gene products. Tandem repeats of epitope tags enhance the detection efficiency of target proteins, which is important for their visualization, especially if they have low-abundance. However, larger tags can potentially interfere with protein function. Therefore, the size of the tag needs to be optimized for each protein of interest. To address this, three tandem genes coding for either HA, PK (V5) and FLAG epitopes, flanked by *Nhe*I and *Xba*I restriction sites were synthesized, and cloned between *Pac*I and *Asc*I sites of the pFA6a-kanMX6 C-terminal tagging plasmid [2] (Fig. 6a and b). PCR-based amplification of the C-terminal tagging plasmid for gene targeting results in addition of a peptide - Arg-Ile-Pro-Gly-Leu-Ile-Asn-Ala-Ser - which acts as a linker between the target protein and the epitopes (Fig. 6A). The resulting plasmids were named pNX3 (plasmid *N* *he*I*-* *X* *ba*I version three).

The pNX3 plasmid series was extended by replacing *kanMX6* with other drug resistance cassettes [*hygMX6*, *natMX6, neoCV, hygML, natCX* and *zeoCV*] or *ura4* ^+^ cassette. The cleavage site of *Nhe*I is compatible with that of *Xba*I, which allows ligation of epitope tags. For example, 3xPK can be sequentially subcloned to generate 6xPK, 9xPK and 12xPK-tagging plasmids. A list of the pNX3 plasmid series created is shown in Table 5. All plasmids were verified by DNA sequencing, and some of the constructs have been used elsewhere [12, 28].

**Table 5:** The C-terminal tagging vectors, pNX3 series

Name	Epitope tag	Marker cassette	Selection
pNX3a-PK3	3xPK	kanMX6	G418
pNX3a-PK6	6xPK	kanMX6	G418
pNX3a-PK9	9xPK	kanMX6	G418
pNX3a-PK12	12xPK	kanMX6	G418
pNX3a-FL3	3xFlag	kanMX6	G418
pNX3a-HA3	3xHA	kanMX6	G418
pNX3a-Myc13	13xMyc	kanMX6	G418
pNX3b-PK3	3xPK	hygMX6	Hygromycin B
pNX3b-PK6	6xPK	hygMX6	Hygromycin B
pNX3b-PK9	9xPK	hygMX6	Hygromycin B
pNX3b-PK12	12xPK	hygMX6	Hygromycin B
pNX3b-FL3	3xFlag	hygMX6	Hygromycin B
pNX3b-HA3	3xHA	hygMX6	Hygromycin B
pNX3b-Myc13	13xMyc	hygMX6	Hygromycin B
pNX3c-PK3	3xPK	natMX6	CloneNat
pNX3c-PK6	6xPK	natMX6	CloneNat
pNX3c-PK9	9xPK	natMX6	CloneNat
pNX3c-PK12	12xPK	natMX6	CloneNat
pNX3c-FL3	3xFlag	natMX6	CloneNat
pNX3c-HA3	3xHA	natMX6	CloneNat
pNX3c-Myc13	13xMyc	natMX6	CloneNat
pNX3d-PK3	3xPK	ura4^+^	-uracil
pNX3d-PK6	6xPK	ura4^+^	-uracil
pNX3d-PK9	9xPK	ura4^+^	-uracil
pNX3d-PK12	12xPK	ura4^+^	-uracil
pNX3d-FL3	3xFlag	ura4^+^	-uracil
pNX3d-HA3	3xHA	ura4^+^	-uracil
pNX3d-Myc13	13xMyc	ura4^+^	-uracil
pNX3a2-PK3	3xPK	neoCV (rev)	G418
pNX3a2-FL3	3xFlag	neoCV (rev)	G418
pNX3a2-HA3	3xHA	neoCV (rev)	G418
pNX3a2-Myc13	13xMyc	neoCV (rev)	G418
pNX3b2-PK3	3xPK	hygML	Hygromycin B
pNX3b2-FL3	3xFlag	hygML	Hygromycin B
pNX3b2-HA3	3xHA	hygML	Hygromycin B
pNX3b2-Myc13	13xMyc	hygML	Hygromycin B
pNX3c2-PK3	3xPK	natCX	CloneNat
pNX3c2-HA3	3xHA	natCX	CloneNat
pNX3c2-Myc13	13xMyc	natCX	CloneNat
pNX3s2-PK3	3xPK	zeoCV (rev)	Zeocin
pNX3s2-FL3	3xFlag	zeoCV (rev)	Zeocin
pNX3s2-HA3	3xHA	zeoCV (rev)	Zeocin
pNX3s2-Myc13	13xMyc	zeoCV (rev)	Zeocin

(rev): neoCV and zeoCV cassettes are inserted in reverse direction

Using PK-tagging plasmids from this series and the *tpz1 Tag* and *Bot* 100 base primer set, we successfully inserted the gene encoding three, six, nine and 12 tandem-repeats of the PK epitope at the end of the coding gene of *tpz1* ^+^ to endogenously express Tpz1 with the PK tagged at the C-terminus (Fig. 6c). Tpz1 is a component of telomere shelterin complex and is a homolog of human TPP1. Tpz1 both positively and negatively regulates telomere length by interacting with different factors [28, 29]. One interacting partner binds to the C-terminal ends of Tpz1, which is required for formation of shelterin to suppress telomere elongation [29–31]. Deletion of *tpz1* leads to telomere deprotection and cell death [31]. As expected, the efficiency of detection of Tpz1 was increased with a number of PK repeats, and the observed difference in band sizes was due to a different number of 3xPK repeats in each sample (molecular weight of the 3xPK is 4.2 kDa). The slower migrating band in each lane, that presumably corresponds to SUMOylated Tpz1 [32], also became readily detectable (Fig. 6c). To determine whether the C-terminal fusion of PK-tags impaired the activity of Tpz1, we measured telomere length of cells bearing Tpz1 fused to a different number of repeats of PK epitopes (Fig. 6d). Whereas three and six tandem PK tags did not affect telomere length homeostasis, nine tandem PK tags led to slightly elongated telomeres. Thus, we conclude that, although larger tags make proteins and their modifications more visible, they may increase the risk of interfering with function.

### Two-step transformation for gene editing at the endogenous locus

A traditional two-step method can utilize the *ura4* ^+^ cassette to introduce point mutations [7]. First, the gene of interest is replaced by the *ura4* ^+^ cassette in a strain where the endogenous *ura4* ^*+*^ gene region has been deleted (*ura4-D18*). Next, the inserted *ura4* ^+^ cassette is replaced with the mutagenized gene allele. The successfully transformed cells, which lost *ura4* ^*+*^, are initially selected using 5-FOA. We previously utilized this method with success to mutagenize the *rap1* gene [12]. This strategy requires the *ura4-D18* mutation in the strain and further epitope tagging may be necessary to determine the stability of the mutant product downstream. To mutagenize essential genes, a diploid strain should be used in which only one allele of the target gene is replaced with the *ura4* ^+^ cassette. However, because the wild type allele has better homology, the targeting construct favors the wild type allele over the deleted allele. Thus, although treatment with 5-FOA can reduce occurrence of false positives, the presence of the wild type allele makes difficult to target the *ura4* ^+^ replaced locus in diploid cells.

To address this issue, we propose a new method, which utilizes anti-selection markers*.* In this method, the gene of interest is replaced with a negative selection cassette from the TK-fusion series. The second step uses a plasmid from the pNX3 series to introduce the desired modification to the gene. Briefly, the gene of interest together with its endogenous promoter is subcloned into a pNX3 plasmid and mutagenized as desired. The TK-fusion cassettes contain the *tef* terminator but not the *tef* promoter. The shared homology between the *tef* terminator and the promoter region of the gene in the pNX3 plasmid and the *TKnat/kan* cassettes permits insertion of the modified gene at the deleted allele *via* homology directed repair.

**Fig. 7:**
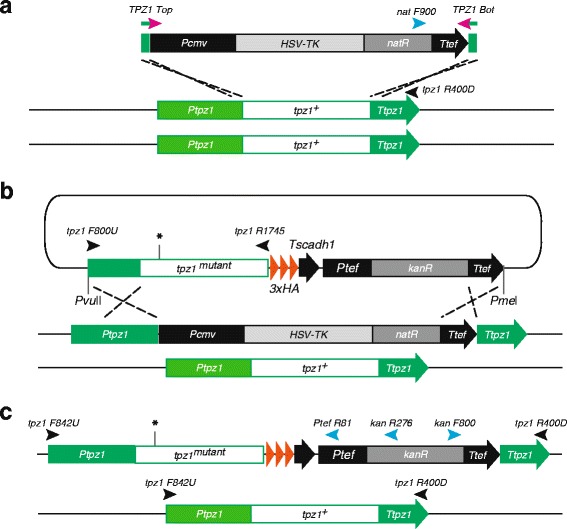
Two-step mutagenesis. Schematic diagram of the two-step gene replacements and induction of point mutation of the *tpz1* gene. List of diagnostic primers is shown in Table 2. **a** PCR based *tpz1* gene deletion by the *TKnatCX* targeting fragment generated using the *tpz1 Top and Bot* 100 base primer set (Table 1). 80–100 base of homologous sequences in the 100 base primer set targets 5’ and 3’ UTR regions of *tpz1* ^+^ of one of endogenous *tpz1* ^*+*^ alleles. Replacement of *tpz1* ^+^ by *TKnatCX* was confirmed by amplification of DNA fragment using diagnostic PCR primers, *nat F900* and *tpz1 R400D*. **b** Replacement of the *TKnat* cassette by the *tpz1* mutant gene. The DNA fragment containing the promoter region and the gene of *tpz1* ^*+*^ was amplified using primers *tpz1 F800U* and *R1745* and cloned between *Eco*O109I and *Nhe*I sites of pNX3a-HA3. The *tpz1* gene was mutated to generate *K75A* mutation using the sight-directed mutagenesis method. *Pvu*II and *Pme*I digested *tpz1(K75A)-3xHA:kanMX6* fragment from pTpz1a4-K75A-HA3 can only recombine with *TKnatCX* deleted *tpz1* allele, as right arm homology *Ttef* sequence is only present in the *TKnatCX* cassette. **c** Integration of *tpz1-K75A* mutation and 3xHA tagging. Correct replacement was confirmed by amplification of DNA fragments using diagnostic PCR primer sets, *kan F800* and *tpz1 R400D,* and *tpz1 F842U* and *Ptef R81* (or *kan R276*)

As an example, we utilized this method to introduce point mutations in the *tpz1* ^*+*^ gene. As *tpz1* ^*+*^ is essential gene, we first deleted one *tpz1* allele in a diploid strain using the PCR-based *TKnatCX* recombination method (Fig. 7a). The cloned *tpz1* ^*+*^ in the pNX3a-3HA plasmid (pNX3a-tpz1-HA3) was mutagenized at the position c.322–323 to give the mutation K75A. The resulting construct, pNX3a-tpz1-K75A-HA3, was digested with the restriction enzymes, and the *tpz1* heterozygous diploid strain was transformed with this targeting construct (Fig. 7b). The resulting transformants were selected by G418 resistance. Most of the positive colonies lost their Nat resistance as successful insertion of the mutant gene would result in removal of the *TKnatCX* cassette. In our experience, success rate of the gene replacement was 96 % (25 cells lost the Nat resistance out of 26 cells resistant to G418). Diagnostic PCR using primer sets shown in Fig. 7c confirmed that all of the transfomants that lost Nat resistance had the correct replacement of *tpz1(K75A)-3xHA:kanMX6* at the *TKnatCM* site. Expression of Tpz1(K75A)-3xHA was confirmed by Western blot. Finally, the resulting heterozygous diploid strain (*h* ^*−*^ */h* ^*+*^ *ade6-M210/M216 tpz1(K75A)-3xHA:kanMX6/+*) was starved to induce sporulation, and the offspring was selected on the G418 to isolate a *tpz1(K75A)* haploid strain. Expression level, function of Tpz1(K75A), and the phenotype of this mutant strain were reported previously [28]. Therefore we showed that this method can be successfully and reliably used to target and modify genes to give stable products.

## Conclusions

We have demonstrated that the replacement of promoter and/or terminator sequences in drug resistance cassettes can eliminate the need to replace existing markers in working strains. This enables sequential genetic manipulations to be performed without making changes to the genetic and epigenetic background of strains. In addition, we have shown that the TK-fusion markers can be used to replace genes and subsequently swapped in order to counter-select for undesirable genotypes. While PCR-based gene targeting remains a powerful and cost-effective method, the modified plasmids and optimized techniques presented in this study would enable researchers to perform their desired genetic manipulations more efficiently and reliably.

## Methods

### Yeast media

All media and supplements were purchased from FORMEDIUM™. Fission yeast were grown at 32 °C.

### Plasmid constructions

All plasmids generated are listed in Tables 3, 4 and 5 and are available from *Addgene* (www.addgene.com). Oligos used for cloning are listed in Table 6. To create pFA6a-*neoCV* and pFA6a-*zeoCV* plasmids, the *CMVneo* and *CMVzeo* cassettes were amplified from pCMVneo and pCMVzeo plasmids, respectively, using primers *Pcmv top-PmeI* and *Tsv40 bqt-BglII*. The *kanMX6* cassette of pFA6a-kanMX6 was digested with *Bgl*II and *Pme*I and replaced with the *CMVneo* and *CMVzeo* cassettes. To create pFA6a-natCX, the *CMV* promoter was amplified from pCMVpuro plasmid using primers *Pcmv top-BglII* and *Tsv40 bot-PmeI*, and the *tef* promoter of the *natMX6* cassette in pFA6a-natMX6 was digested with *Bgl*II and *Nco*I (blunt ended) and replaced by *Bgl*II and *Kpn*I digested (blunt ended) *Pcmv* PCR fragment. To create pFA6a-hygMV, the terminator of the *sv40* was subcloned from pSV2-hyg plasmid. pSV2-hyg was digested with *PfM*I and *Hpa*I and self-ligated to remove the gene encoding large tumor antigen, and the *hyg* resistant gene with *Tsv40* was cloned using primers, *Hyg* ^*R*^ *top-HindIII* and *Tsv40 bot-PmeI*. The *tef* terminator along with a part of the *hyg* resistant gene of pFA6a-hygMX6 was replaced with the *hyg* ^*R*^ *–Tsv40* PCR product using *Sac*II and *Pme*I sites, and resulting plasmid was named pFA6a-hygMV. To replace the terminator of the *hygMX6* cassette with the *S. cerevisiae LEU2* gene, the terminator of *LEU2* was cloned from the *S. cerevisiae* genomic DNA using primers, *Tscleu2 top-ClaI-PstI*, and *Tscleu2 bot-PmeI*, and inserted between *Pst*I and *Cla*I sites of pFA6a. The *tef* promoter and the *hyg* resistant gene were cloned using primers, *Amp rev* and *Hyg bot-NheI-ClaI*, and inserted at *Bgl*II-*Cla*I sites of pFA6a. The resulting plasmid pFA6a-Ptef-hyg was digested with *Cla*I and the *LEU2* terminator was inserted to generate pFA6a-hygML.

**Table 6:** Oligos used for cloning and plasmid construction

Name	Sequence
*Pcmv top-PmeI*	atcatgaccggtttaaacCGTTACATAACTTACGGTAA
*Pcmv top-BglII*	atcaccggtgctagcagatctcgcgaCGTTACATAACTTACGGTAA
*Tsv40 bot-BglII*	atccctaggctgcagatctGCAGTGAAAAAAATGCTTTA
*Tsv40 bot-PmeI*	agctgtacagatatcgagctcgtttaaacGCAGTGAAAAAAATGCTTTA
*Hyg top-HindIII*	atcctgaagcttacatgtccATGGGTAAAAAGCCTGAACTCACCGCGACG
*Hyg bot-NheI-ClaI*	atcgattagctagcTACTTCTACACAGCCATCGGTCC
*Tscleu2 top-ClaI-PstI*	atcgattagctgcagtAATCCTTGCTTAAAAAGATTCTCT
*Tscleu2 bot-PmeI*	agctgtacagatatcgagctcgtttaAACTCCATCAAATGGTCAGG
*HSVtk top-SpeI-NcoI-HIndIII*	atcaagcttccatggaaactagtGCGTTCGACCAGGCTGCGCGTT
*HSVtk bot-XmaI-NcoI*	atcgattaacccatggccCGGGCAAACGTGCGCG
*Padh1 top-PmeI*	tcaggcccttgaattcgtttaaacCCTACAACAACTAAGAAAATGGCTATCATGCGGAAG
*Padh1 bot-HindIII-NheI*	atcgctagcAAgcTTCTCTTGCTTAAAGAAAAGAAAAGCGAAGGCACCTGTCCACCACCC
*100 bp-bot*	GAATTCGAGCTCGTTTAAAC
*Tscadh1 For-AscI*	tgaggCGCGCCACTTCTAAATAAGCG
*Tscadh1 R110-BglII*	aagatctCCTAGCGGATCTGCCGGTAG
*Amp rev*	CGACACGGAAATGTTGAATACTC
*ori-AflIII*	CCTTTTGCTGGCCTTTTGCTCACAT
*lox71-Padh1 top-BglII*	acggttaattaagatctaccgttcgtatagcatacattatacgaagttatactagtcatatgCCTACAACAACTAAGAAAATGGCTATCA
*lox66-Ttef bot-PmeI*	atcgaattcgagctcgtttaaactaccgttcgtataatgtatgctatacgaagttataggcctaGGATGGCGGCGTTAGTATCG
*nls-Cre top-XbaI*	atttaattaactctagaTGTACTCCACCAAAGAAGAAGAGAAAGGTTGCCcctagcATGGCCAATTTACTGACCGTACACCA
*Cre bot-XbaI*	aggctagcggcgcgccTCAtctagaATCGCCATCTTCCAGCAGGC
*ura4 F530U-BglII*	ggcatagatctgagctcgatatcAAGCTTAGCTACAAATCCCACTGGCTATATGT
*ura4 R190D-PmeI*	ttacctgagctcgtttaaacAAGCTTGTGATATTGACGAAACTTTTTGACATCTAAT
*leu1 F130U-BglII*	aagcttagatctCCATACGATATCCCAATCTGTAG
*leu1 R200D-PmeI*	atcgagctcgtttaaacGGATGTCGTAAATCAATTCCATGC
*arg3 F130U-BglII*	aagatcTCGGCTATATGCAATCTCAC
*arg3 R130D-PmeI*	aagtttaaacAGCCTGTGTCCTGCGCATA
*ade6 F300U-BglII*	aagatcTAAGGTATAACGACAACAAACGTTGC
*ade6 R150D-PmeI*	aagtttaaaCTGCTTCACAGCACATTATTCAGGATTCTT
*aur1 F333U-BglII*	aagtcagcggcgcgccaagatctGTCAGGAAAGCTTTTTTGCCTCT
*aur1 R280D-PmeI*	agaattcgagctcgtttaaaCAAATACCTATACATCACACTGGAA
*3xHA top*	atccccgggttaattaacgctagcatgTACCCATACGATGTTCCTGACTATGCGGGCTACCCGTATGACGTGCCGGACTATGCAGGAGCCTATCCTTATGACGTTCCAGA
*3xHA bot*	aggctagcggcgcgccTCAtctagaACCAGCGTAATCTGGAACGTCATAAGGATAGGCTCCTGCATAGTCCGGCACGTCATACGGGTAGCCCGCATAGTCAGGAACATCG
*3xPK-H6 top*	ggatccatatgTTAATTAACGCTAGCGGTAAGCCTATTCCTAACCCTCTTTTGGGTCTCGATTCTACAGGTGGAAAACCAATCCCCAACCCACTCCTCGGCCTTGACTCAACTGGCAAAC
*3xPK-H6 bot*	agatctggcgcgcctaggatggtgatggtgatgatgtctagaGGTACTATCTAATCCAAGTAAAGGATTTGGTATGGGTTTGCCAGTTGAGTCAAGGCCGAGGAGTGGGTTGGGGATTGG
*3xFlag top*	taacgctagcatgGGTGACTACAAGGACGACGATGACAAGGGAGATTACAAAGATGACGACGATAAGGACTACAAGGACGACGATGACAAGtctagaTGAgg
*3xFlag bot*	cgcgccTCAtctagaCTTGTCATCGTCGTCCTTGTAGTCCTTATCGTCGTCATCTTTGTAATCTCCCTTGTCATCGTCGTCCTTGTAGTCACCcatgctagcgttaat
*tpz1 F800U-PvuII-EcoO*	atcaggccctcagCTGTTCAGCACGGTACCAAG
*tpz1 R1745-NheI*	gttgcagctagcGCTTTTGTTTCGAAACTCCTCTAT

Uppercase at 3’ end of oligo anneals to target and lowercase at 5’ end is a linker containing restriction sites and additional sequence

To create pFA6a-TKanMX6 and pFA6a-TKnatMX6, the *TK* gene was cloned using primers, *HSVtk top-SpeI-NcoI-HindIII* and *HSVtk bot-XmaI-NcoI*, digested and inserted at *Nco*I site of pFA6a-kanMX6 and pFA6a-natMX6. To create pFA6a-HyTKMX6, the *TK* PCR product was digested with *Nco*I and *Xma*I (blunt ended) and replaced *nat* ^*R*^ gene of pFA6a-natMX6 using *Nco*I and *Sph*I (blunt ended) sites. The resulting plasmid was named pFA6a-HSVTKMX6. pFA6a-hygML was digested with *Nhe*I and *Pme*I, and *Tscleu2* was replaced with the *Spe*I and *Pme*I digested *HSV-TK-Ttef* fragment from pFA6a-HSVTKMX6. To create pFA6a-TKnatCX, the *CMV* promoter was cloned from pCMVzeo using primers *Pcmv top-BglII* and *zeo R115* and digested with *Bgl*II and *Msc*I. TKnatMX6 was digested with *Bgl*II and *Spe*I (blunt ended), and the *tef* promoter was replaced with the *CMV* promoter.

The *adh1* promoter was amplified from *S. pombe* genomic DNA using primers, *Padh1 top-PmeI* and *bot-HindIII-NheI,* and cloned into pJET vector (ThermoFisher). pJET-Padh1 was digested with *Pme*I and *Cla*I (within pJET), and the *natCM* cassette of pFA6a-natCM was replaced with *Padh1* using *Nru*I and *Cla*I digest to generate pFA6a-Padh1. To create pFA6a-TKanAX and pFA6a-TKnatAX, pFA6a-TKanMX6 and pFA6a-TKnatMX6 were digested with *Spe*I, and the *TKan* and *TKnat* genes were inserted at *Spe*I site of pFA6a-Padh1 in forward direction. To create pFA6a-HyTKAX, the *HyTK-Ttef* fragment was amplified from pFA6a-HyTKMX6 using primers, *Hyg top-HindIII* and *100 bp-bot*, and cloned into pFA6a between *Hin*dIII and *Pme*I sites, and *Padh1* from pFA6a-Padh1 was inserted at *Hin*dIII site in forward direction.

The Cre expression plasmid was created as follows. The *CMV* promoter was isolated from pAUR224 (TAKARA/Clontech) using *Tsp*45I (blunt ended) and *Hin*dIII, and was inserted at *Nde*I (blunt ended) and *Hin*dIII site of pFA6a-13xmyc-hygMX6. The three tandem HA epitope encoding DNA fragments (*3xHA*) were synthesized by annealing of oligos, *3xHA top* and *bot*, followed by DNA polymerase reaction. The 3xHA DNA fragment was generated by digest with *Pac*I and *Asc*I and replaced 13xmyc gene. The Cre recombinase encoding gene was cloned using primer *nls-Cre top-XbaI* that includes *sv40* nuclear localization signal (NLS) at 5’ linker and *Cre bot-XbaI*, and was digested and inserted at XbaI site downstream of 3xHA. The resulting plasmid carried the cassette expressing 3xHA-nls-Cre from the *CMV* promoter and was named pNXVa-HA3nlsCre. The DNA fragment containing the *S. pombe* replication origin, *ars1*, was isolated by *Avr*II digest of the *S. pombe* plasmid pKAN1. pFA6a-kanMX6 was digested with *Eco*RI and *Sap*I, and the *ars1* containing *Avr*II fragment was inserted following blunt ending of the cleaved sites. The resulting plasmid that carried *kanMX6* and *ARS1* was named pNXRa. The Cre expression cassette was isolated by *Eco*O109I and *Bgl*II digest and inserted into pNXRa to generate pNXRVa-HA3nlsCre.

Auxotrophic markers *ura4* ^*+*^, *leu1* ^*+*^, *arg3* ^*+*^ and *ade6* ^*+*^ were cloned from *S. pombe* genomic DNA using corresponding primer sets listed in Table 6, and cloned into pJET vector. To generate Cre-expression vectors with various selection cassettes, auxotrophic markers *leu1* ^+^, *arg3* ^+^, *ura4* ^+^and *ade6* ^+^ and drug selection markers *TkanAX*, *hygMX6*, *HyTkAX*, *natMX6*, *TKnatAX* and *aur1* ^*R*^ from the plasmid pAUR224 were amplified with primers containing with *Bgl*II and *Pme*I sites and cloned into pJET vector (ThermoFisher). The cloned cassettes were digested with *Bgl*II and *Pme*I and replaced with *kanMX6* of pNXRVa-HA3nls-Cre. For the systematic reason, the drug resistant cassettes for G418, hygromycin B, clonNat, and aureobasidin A and auxotrophic markers for uracil, arginine, leucine, and adenine were assigned as ‘a’, ‘b’, ‘c’, ‘j’, ‘d’, ‘g’, ‘h’ and ‘k’, respectively. Through the cloning process, we found point mutation (G201T) in the Zeocin resistant gene that substitutes Tryptophan at the position 67 to Cysteine (W67C), but the cassette retained resistance to Zeocin (Fig. 1b).

The series of the C-terminal tagging plasmids, pNX3 was constructed as follows. The terminator of the *S. cerevisiae adh1* was cloned from the genomic DNA using primers, *Tscadh1 For-AscI* and *Tscadh1 R110-BglII*, and inserted into pNXRa after digestion with *Asc*I and *Bgl*II. *ARS1* sequence was removed by digest of pNXRa with *Eco*RI and *Avr*II and ligation following blunt ending of the cleaved sites. Unlike pFA6a C-tag plasmids, this resulting plasmid, named pNX3a, lacks *I-Sce*I after *Tadh1* and *Cla*I, *Eco*RV, *Spe*I, *Sfi*I, *Not*I, *Sac*II, *Hpa*I and *Sap*I after kanMX6. To generate pNX3a-HA3, the *3xHA* fragment was produced by digest as mentioned above and inserted into pNX3a following *Pac*I and *Asc*I digest. Likewise, the three tandem *PK* fragment (3xPK) was generated by annealing of oligos, *3xPK-H6 top* and *bot*, followed by DNA polymerase reaction. To generate pNX3a-PK3, the 3xPK fragment was obtained by digest and replaced 3xHA of pNX3a-HA3 after *Pac*I-*Xba*I digest. Three tandem *FLAG* fragment (3xFL) was generated by annealing of oligos, *3xFlag top* and *bot*, followed by 5’ end phosphorylation. To generate pNX3a-FL3, the 3xFL fragments were inserted into pNX3a after *Pac*I and *Asc*I digest. The *kanMX6* cassette was replaced by other selection markers using *Bgl*II and *Pme*I digest. All generated plasmids for the C-terminus tagging are listed in Table 5.

### PCR amplification for gene targeting fragments

For amplification of the deletion fragments using pFA6a series, the primer set (*Top* and *Bot*) was used. *Top* primer is composed of 80–100 base forward sequence upstream of the start codon of the target gene at the five prime end and the vector annealing sequence CGGATCCCCGGGTTAATTAA at the three prime end. *Bot* primer is composed of the complement sequence of downstream 80–100 base sequence after the stop codon of the target gene at the five prime end and vector annealing sequence GAATTCGAGCTCGTTTAAAC at the three prime end.

For C-terminus tagging using pNX3 series, the C-tag specific Tag primer and the deletion Bot primer were used. The annealing sequence of Tag primer is the same as Top primer for deletion but targeting sequence is 80–100 base upstream from the end of the gene excluding the stop codon. These oligos were synthesized by Integrated DNA Technologies Inc. (IDT) using standard desalting purification service. Details of primer design have been described previously [2].

Three hundred microliter of PCR mixtures contained 30 μl of 10xbuffer I, 6 μl of 10 mM dNTPs and 1.5 μl of long template polymerase mixture (Roche) with 0.75 μl of pFA6a or pNX3 template vector (approximately 50–500 ng/μl, obtained using standard miniprep kit), and 1.5 μl each of 100 μM primers. Premix was aliquoted into 50 μl and subjected to PCR [94 °C 30 sec, 50 °C 2 min, 68 °C 5 min for 5 cycles, followed by 94 °C 30 sec, 55 °C 1 min, 68 °C 5 min for 30 cycles, finishing with 68 °C 10 min]. PCR products were combined, ethanol precipitated, and reconstituted in 10 μl of TE (10 mM Tris–HCl 1 mM EDTA pH8.0).

### PCR-based gene targeting

Fission yeast transformation was performed as described [2] with minor changes. Logarithmically growing cells ~10^7^ cells/ml (O.D_600_ = 0.5–0.8) were harvested, and washed once with LiOAc solution (100 mM Lithium Acetate pH7.5 in TE). Cell pellets were suspended in equal volume of LiOAc solution to make competent cells. 50 μl of the competent cells were mixed with the gene targeting PCR product, 130 μl of 40 % PEG solution (40 % of PEG4000 in LiOAc solution) was added to the cells and mixed gently by tapping. Cells were incubated at 32 °C for 1–4 h. After adding 21.5 μl of DMSO, and the cells were heat-shocked at 42 °C for 5 min, precipitated briefly and washed once with YES media. The transformed cells were directly plated on EMM media for auxotrophic selection or YES/EMM media for Aureobasidin B (TAKARA/Clontech) selection. For other drug selection, transformed cells were recovered in YES media for 4 h in shaking incubator or overnight on the plate, and transferred to YES plate containing G418 (FORMEDIUM), Zeocin (Invivogen), Hygromycine B (FORMEDIUM and Roche) or ClonNat/Nourseothricin (WERNER BioAgents). YES can be replaced with PMG media.

### Cre-loxP counter selection

To remove *loxP*-flanked *TK* fusion cassette, cells were transformed with any Cre expression vector (see transformation procedure in previous section). After heat- shock process, cells were directly plated on FdU containing YES plate. FdU-resistant colonies were re-streaked on a fresh FdU plate to isolate a single colonies. Loss of *TK* fusion cassette and the Cre expression vector were confirmed by sensitivity to the drugs. Alternatively, pNXRVj-HACre was used for Cre expression, the transformants carrying Cre vector were selected by resistance to aureobasidin B. The resistant colonies were re-streaked on YES plates, and colonies that lost the drug resistance were isolated.

### Southern blot

Telomere Southern blotting was performed as described previously [28]. Genomic DNA was prepared 2 weeks after generation of the strains unless indicated. Equal amounts of *Eco*RI digested DNA fragments were separated on a 1 % agarose gel and subjected to Southern blotting with a telomere probe.

## Abbreviations

5-FOA: 5-Fluoroorotic acid
CMV: Cytomegalovirus
CRISPR: Clustered regularly interspaced short palindromic repeats
dNTP: deoxynucleotide
DSB: Double-strand break
dTMP: deoxythymidine-5-monophosphate
dTTP: 2’-deoxythymidine-5-triphosphate
ENT: Equilibrative nucleoside transporter
FdU: 5-Fluoro-2’-deoxyuridine
FdUMP: 5-Fluoro-2’-deoxyuridine-5-monophosphate
HA: Hemagglutinin
HSV: Herpes simplex virus
HU: Hydroxyurea
*loxP*: Locus of cross-over P1
MCS: Multiple cloning sites
NLS: Nuclear localization signal
sv40: Simian virus 40
TK: Thymidine kinase
